# Molecular insight into histone methylation as a novel target for oral squamous cell carcinoma: future hope in personalised medicine

**DOI:** 10.7150/jca.103243

**Published:** 2025-01-27

**Authors:** Peramaiyan Rajendran, Monisha Prasad, Enas M Ali, Ramya Sekar, Abdullah M AlZahrani, Mohmed Isaqali Karobari, Marwa Azmy M. Genena, Basem M Abdallah

**Affiliations:** 1 Department of Biological Sciences, College of Science, King Faisal University, Al-Ahsa, 31982, Saudi Arabia.; 2 Centre for Global Health Research, Saveetha Medical College and Hospitals, Saveetha Institute of Medical and Technical Sciences (SIMATS), Saveetha University, Chennai 602105, TN, India.; 3 Department of Oral & Maxillofacial Pathology and Oral Microbiology, Meenakshi Ammal Dental College and Hospital, Meenakshi Academy of Higher Education and Research (Deemed to be University), Chennai, Tamil Nadu, India.; 4 Department of Conservative Dentistry and Endodontics, Saveetha Dental College and Hospital, Saveetha Institute of Medical and Technical Sciences, Saveetha University, Chennai 600077, Tamil Nadu, India.

**Keywords:** Oral cancer, Histone methylation, Cancer, OSCC, Biomarkers, therapeutic target

## Abstract

Oral squamous cell carcinoma (OSCC) is the most prevalent type of malignant epithelial neoplasm that affects the oral cavity. It has been a significant health concern in many countries for a long time since it was usually treated with surgery, radiation, and/or chemotherapy. Drug resistance is the primary issue in patient populations and scientific research, which promotes OSCC tumour cell invasion and migration. Thus, identifying highly specific therapeutic targets could be the potential approach for more successful treatment of OSCC. It is still challenging to understand the genetic causes of oral carcinogenesis due to its highly varied clinic-pathological parameters. It is important to remember that signaling channels and complexes that affect chromatin accessibility control gene expression, which in turn affects cell development and differentiation. Histones undergo post-translational alteration to give this platform. Understanding the processes of gene regulation through histone methylation and its modifications could enhance the early detection, prognostic prediction, and therapy of OSCC. To be properly used as a therapeutic target, histone methylation in OSCC requires more investigation. This review details the dysregulated histone methylation and the modifying enzymes linked to the development and aetiology of OSCC. Furthermore, the part that lysine methylation plays in cell migration, chemo-resistance, and OSCC invasion is also investigated.

## Introduction

Oral cancer encompasses a broad range of malignancies that can develop in the salivary glands, pharyngeal regions, and oral cavity. However, the term is sometimes used interchangeably with OSCC, the maximally prevalent subtype of all oral neoplasms. OSCC affects the epithelial lining of various areas such as the paranasal sinus, pharynx, larynx, nasal cavity, and oral cavity. Research findings consistently indicate that 90% of all oral neoplasms are OSCC 1. India bears a significant burden of oral cavity cancer, surpassing the rates observed in many Western countries. In India, almost 70% of patients were diagnosed at locally advanced stages of cancer (AJCC Stage III-IV), which drastically lowers the chance of recovery. The grim reality is reflected in the five-year survival rates, which typically hover around a mere 20% for these patients. Compounding the challenge, individuals diagnosed with OSCC in India often grapple with poor overall health, inadequate nutritional status, and other underlying health conditions, making curative treatments exceptionally challenging.

The high incidence of oral squamous cell carcinoma (OSCC) in India presents a serious public health concern. Oral cancer accounts for nearly one-third of all worldwide cases, with the case and death rates rising dramatically in India from 1990 to 2021. The study has strong state and gender disparities in this regard and forecasts to continue growing by 2031. Targeted preventive, early detection, and mitigation strategies are critical steps toward curbing this trend 2. The most commonly afflicted areas in both genders are the buccal mucosa and gingivobuccal sulcus, highlighting the disease's major effects on the oral cavity 3.

The prognosis of OSCC is influenced by a complex interplay of factors, including genetics, diet, and environmental elements 4. Genetic alterations, such as gene amplification, deletion, and mutation, can activate oncogenes or suppress tumor suppressor genes, leading to the development of oral premalignancy and OSCC. Lifestyle factors like a sedentary lifestyle, smoking, radiation exposure, and exposure to infectious agents further increase the risk of oral and oropharyngeal cancer by affecting key pathways like Akt, NFkB, and Wnt. These alterations in genes associated with tumorigenesis, such as tumor suppressor genes (p16, TP53, and pRb) and proto-oncogenes (EGFR, Ras, and myc), significantly impact the origin and progression of OSCC malignancies 4,5. Global Burden of Diseases, Injuries, and Risk Factors Study 2019 estimates that OSCC accounts for about 377,000 new cases and approximately 177,000 deaths globally each year, making it a major public health concern. Risk factors include alcohol intake, tobacco usage, and chewing betel quid contribute to the occurrence, which is highest in South and Southeast Asia. The prevalence of OSCC is higher in low-income nations, which emphasizes the need for focused public health campaigns, efficient screening programs, and treatment accessibility. To improve health outcomes and provide priority to research in this field, it is imperative to address the worldwide burden of OSCC 6,7.

Epigenetics, a term referring to genetic abnormalities not caused by variations in the DNA sequence is a crucial part in the progress and spread of cancer through the regulation of gene expression. Several studies reported the factors affecting DNA methylation, histone alterations, microRNA expression, and nucleosome orientation in cancer their role in the genesis and development of the disease 8. Epigenetic modifications control gene expression through fundamental processes such as methylation, acetylation, phosphorylation, and ubiquitylation of histone proteins, as well as DNA methylation. Epigenetic enzymes recognize these modifications, altering the structural conformation of chromatin. This can either compact the chromatin to restrict transcription factor binding or open it to allow binding, thereby influencing gene transcription. These processes ultimately regulate the expression of genes involved in cellular functions 9-11.

Recent research has brought attention to newly discovered epigenetic targets, such as unique histone modifications and the part played by long non-coding RNAs (lncRNAs) in the development of OSCC. The intricacy of chromatin remodeling in OSCC has become more apparent with the development of our knowledge of histone methylation, particularly the interaction between methylation and acetylation marks. Furthermore, a new perspective on how epigenetic modifications promote tumor initiation and metastasis has been provided by the discovery of novel non-coding RNAs and their impact on chromatin state and gene expression 12. Furthermore, the ability to precisely modify particular histone marks has been made possible by recent advancements in CRISPR-based epigenome editing technologies, which presents the possibility of personalized therapeutic approaches for OSCC. With the aid of these technologies, researchers may alter the histone marks at certain loci, shedding light on the ways in which histone methylation controls the expression of important genes involved in the development and spread of cancer and creating new avenues for the development of tailored epigenetic treatments for OSCC 13

Lysine-specific methyltransferase 2D (KMT2D) was reported to be frequently altered in OSCC. KMT2D knockdown in patient-derived cells reduced CD133 and β-catenin expression in OSCC, affecting cells to form colonies, exhibit motility and invasiveness in animal and cell line models, and thus, slowing the cancer growth. This has opened new avenues in cancer treatment, where epigenetic medications are being explored in combination with endocrine therapy, cytotoxic chemotherapy, targeted medicines, radiation, and immunotherapy. Among these, DNA methyltransferase (DNMT) inhibitors and histone methylation inhibitors showed significant promise in treating cancer. Advances in targetable epigenome-editing techniques enable the direct attribution of transcriptional and functional consequences to locus-specific chromatin changes, offering valuable insights into the biology of oral cancer and paving the way for novel therapeutic approaches in precision medicine 14. These advances in targetable epigenome-editing techniques enable direct attribution of transcriptional and functional consequences to locus-specific chromatin modifications. The significance of histone methylation in the biology of oral cancer is summarized in this review as a new therapeutic mark in precision medicine.

## Overview of histone modifications

Gene expression regulation requires perfect coordination between several proteins and DNA. The eukaryotic nucleosome (the fundamental building block of chromatin) is surrounded by a 147-base-pair stretch of DNA that has two copies of each core histone, including H2A, H2B, H3, and H4. The characteristic side chain, or tail, of each of the mostly globular histone proteins is tightly packed with basic lysine and arginine residues. The connections among the bulk of the four-core histone protein helix C-terminal domains result in the octameric column-like structure that surrounds the DNA 15. The majority of the structurally unknown but evolutionarily conserved "tail" domains make up the remaining core histone. Enzymes that carry out crucial post-translational modifications (PTMs) for epigenetic control have easy access to these tail domains. This adaptable histone tails, in particular, are hotspots for unique histone modifications because they are abundant in hydroxyl containing Ser/Thr/Tyr and basic Lys/Arg amino acids groups. Covalent PTMs can quickly change the tails that stick out from the nucleosome's surface. PTMs also influence a number of biological processes by activating or inactivating genes and alter the chemical composition of histones by adding or deleting chemical groups 16. Histone modifications are essential epigenetic regulatory elements with a significant impact on numerous cellular processes. For instance, these histone modifications influence chromatin construction and aid as binding sites for a variety of transcription factors, including DNA/histone-modifying enzymes, chromatin remodels, histone chaperones, and general transcription factors. Numerous cellular progressions, including heterochromatin compaction, cell cycle, DNA replication and repair, gene expression, and many others, depend on these histone modifications 17-20.

**Figure 1:**
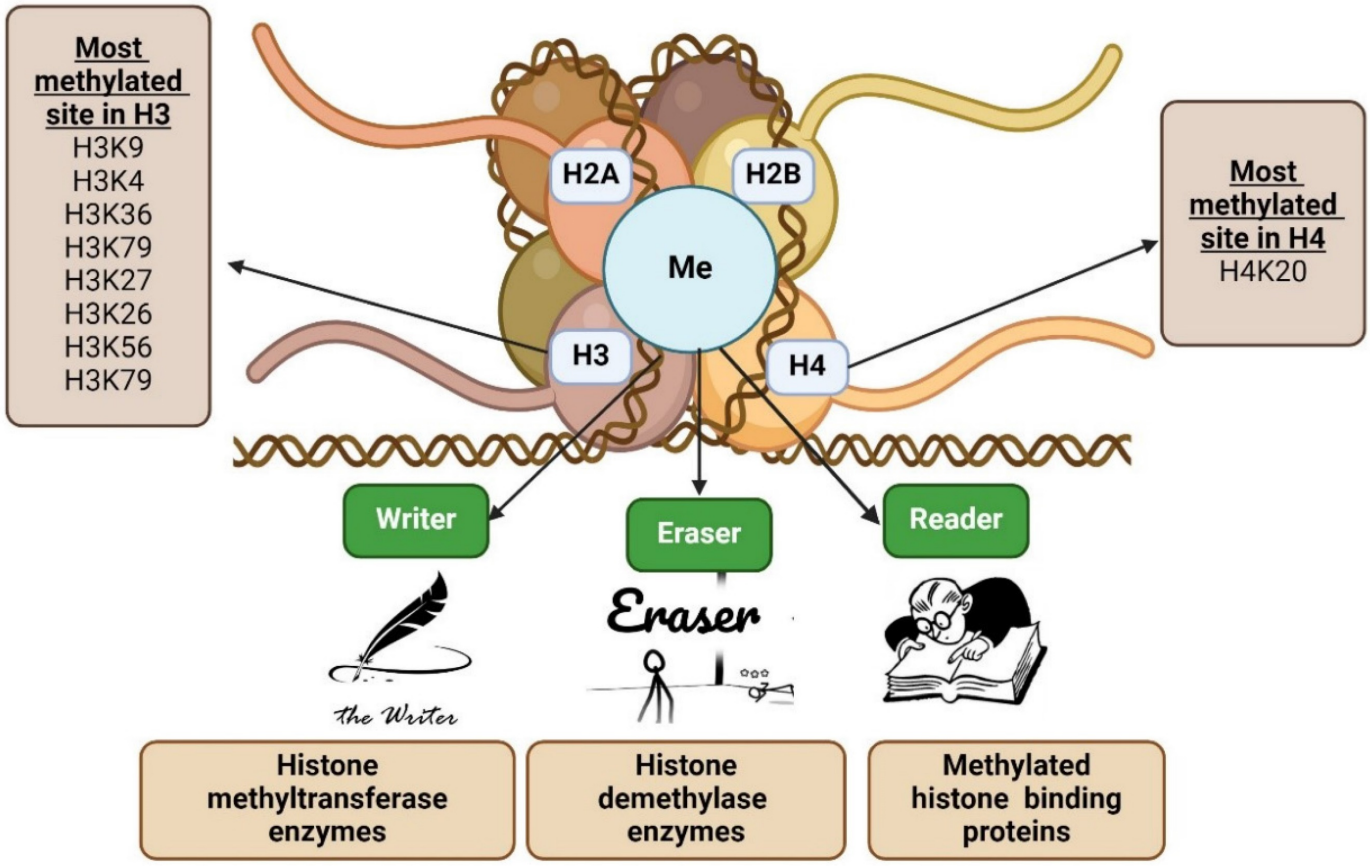
** Overview of histone methylation.** Histone methylation happens more frequently in flexible histone tails due to the presence of basic amino acids. H2A, H2B, H3, and H4 are among the extended-tail histone proteins that are methylated during nucleosome modification. Histone methylation typically affects the transcriptional activation or inhibition of downstream genes in H3 and H4 regions, mostly via the writer, eraser, and/or reader.

The majority of post-translational modifications to histones occur in one of two ways: covalently or non-covalently. Acetylation of lysine residues is the most common type of covalent histone modification. Other covalent modifications include methylation of arginine and lysine residues, sumoylation, ubiquitination of lysine residue at the histone tail, phosphorylation of serine and threonine residues, and ADP ribosylation. Histone variations and nucleosome remodelling are examples of non-covalent histone modifications 21-23. The most frequent epigenetic alteration, which can either help or impede gene function contingent on the conditions. H2A, H2B, H3, and H4 are only a few examples of the extended-tail histone proteins that are the targets of methylation in nucleosome modification. In H3 and H4 areas, transcriptional stimulation or inhibition of downstream genes is frequently associated with histone methylation. H3K4, H3R8, H3R17, H3K26, H3K36, H3K79, H4K12, and H4R3 methylation can initiate gene transcription, but methylation of particular histones (H4K5, H4K20, H3K9, H3K27, and H3K56) controls gene transcription, exposing the intricacy of epigenetic aspect of histone (Figure.1) 24-26. Furthermore, this process is a reversible methylation that modifies normal cell metabolism in a healthy physiological system using particular type of enzyme 27. Histone methyltransferases are enzymes that promote the monomethylation, dimethylation, or trimethylation of histones, while histone demethylases are responsible for the demethylation of histones 24. While histone demethylases, such as lysine-specific demethylase 1 (LSD1), are in charge of controlling histone demethylation, histone lysine methyltransferases (KMTs) are in charge of adding the methyl group from S-adenosylmethionine to the N-terminal tails of the lysine residues found on histones 28. Histidine (His or H), arginine (Arg or R), and lysine (Lys or K) are the most common histone methyl acceptors (His or H) 29,30. In addition, the neighbouring amino-acid sequence and methylation status affect how methyl-histone recognition proteins identify particular methyl-lysine's. These modular protein domains are involved in chromatin-regulatory functions that are able to identify particular changed histone species and translate them into different downstream biological effects.

Multiple malignancies and developmental abnormalities are linked to dysregulation of any of this histone methylation 31. For instance, in a rat tumour model the activity of mixed-lineage leukaemia 1 (Mll1) and genome-wide elevated H3K4 trimethylation at promoters are both increased in the tumor-propagating cells. High Wnt/b-catenin and Mll1 were linked to high H3K4me3 at promoters in mouse tumors, confirming the role of H3K4 methyltransferase activity in the transcriptional response to Wnt/β-catenin signals in the transcription of salivary gland of squamous cell carcinoma in mice 32. The pathophysiology of malignancies may be influenced by altering the balance of these histone modifications of gene expression 33,34. This element of epigenetics regulates gene expression in a coordinated and interdependent manner. These epigenetic mechanisms currently dominate transcriptional regulation and associated dysregulation in cancer, despite initially receiving less research attention. The transgenerational implications, however, are mainly unknown. A critical regulatory framework for actions including gene expression, DNA replication and repair, chromosomal condensation and segregation, and apoptosis is provided in malignancies by the post-translational modification of histones. According to Audia and Campbell 17, this dysregulation can lead to the incorrect activation of oncogenes or, in the opposite situation, the inappropriate inactivation of tumour suppressors. In addition, the genetic underpinnings of the epigenetic changes seen in cancer are becoming more and more understood and recognized. Particularly, because of its importance in both the pathological and physiological states of OSCC, histone methylation has drawn more and more attention. New targets for the earlier detection and treatment of OSCC can be found in the abnormal histone methylation regulation.

## Histone methylation in OSCC progression

Tumor cells primarily exhibit anomalies in cellular identity, dysregulation of gene expression, and responses to internal and external cues 35,36. As a matter of fact, these epigenetic systems are tightly regulated and controled adult life as well as embryonic development, and their dysregulation has linked to a variety of cancer 18. Large-scale cancer genome sequencing projects have revealed that chromatin protein mutations are present in nearly half of all human malignancies 37. Additionally, this histone methylation is also having an impact on the functional traits that normal cells acquire during the process of tumour formation, which are the catalysts for carcinogenesis. The traits of cancer are among these alterations, along with ways to strengthen the proliferative signal, thwart growth inhibitors, permit replicative immortality, avoid cell death, and stimulate angiogenesis. They can also lead to invasion and metastasis, cause genome instability and mutation, induce inflammation that promotes tumor growth, reprogramme energy metabolism, and prevent immune destruction 38. Epigenetic changes are necessary for reorganisation of chromatin from a condensed form to a transcriptionally active one. This enables genomic DNA to interact with the transcription-controlling proteins, which in turn regulates gene expression. Histone tail modifications thus directly affect chromatin condensation, which can occur in a heterochromatin or euchromatin configuration. Moreover, the H3K4 histone methylation pattern is linked to OSCC malignancy.

In contrast to normal tissues, OSCC has a higher prevalence of transcriptionally inactive H3K4me2 histones while the opposite was true of H3K4me3 activating alterations 39. This epigenetic reprogramming is facilitated by a number of chromatin-modifying enzymes and is implicated in numerous stages of the evolution of cancer. One of the most often altered genes in OSCC is the histone lysine methyltransferase KMT2D. By maintaining the myocyte-specific enhancer factor 2A (MEF2A)-mediated transcriptional activity of Catenin Beta 1 (CTNNB1), KMT2D overexpression supports stem-like characteristics and Wnt/-catenin signalling in OSCC cells 14. Lysine demethylases 5C (KDM5C) and KDM6A were identified as cancer driver genes that support histone demethylation and hypoxia reprogramming in cancer metabolism 40-42. This was supported by epigenomic therapy using low doses of methylation and acetylation inhibitors like HMTis (3-deazaneplanocin A: DZNep), HMTis (5-Azadeoxycytidine: 5-Aza-dC), and HDACis (trichostatin A: TSA) on human OSCC cells, which showed similar effects on OSCC by histone methylation compared to acetylation inhibitors. The significant effects of histone methylation on OSCC, opening the door to further investigation to study the potential use of histone methylation as an epigenetic therapy target and treatment aspect for OSCC 43,44. In addition, phosphorylation, acetylation, ubiquitination, methylation, sumoylation, and ADP-ribosylation as well as other post-translational modifications to histone at the amino-terminal ends can all have a substantial effect on oral cancer. It's crucial to realize the elements involved in histone methylation, such as histone methyltransferase-HMTs (writer), histone methylation detecting proteins (readers), and histone methylation-regulatory proteins (erasers), before contemplating the therapeutic features of histone methylation (histone demethylases-HDMs) 45,46. These components will be thoroughly examined to show how the epigenetic phenomena may offer hope for cancer therapies. As a result, all of these components are examined in terms of OSCC in this review.

## Regulators of histone methylation in OSCC

**Figure 2:**
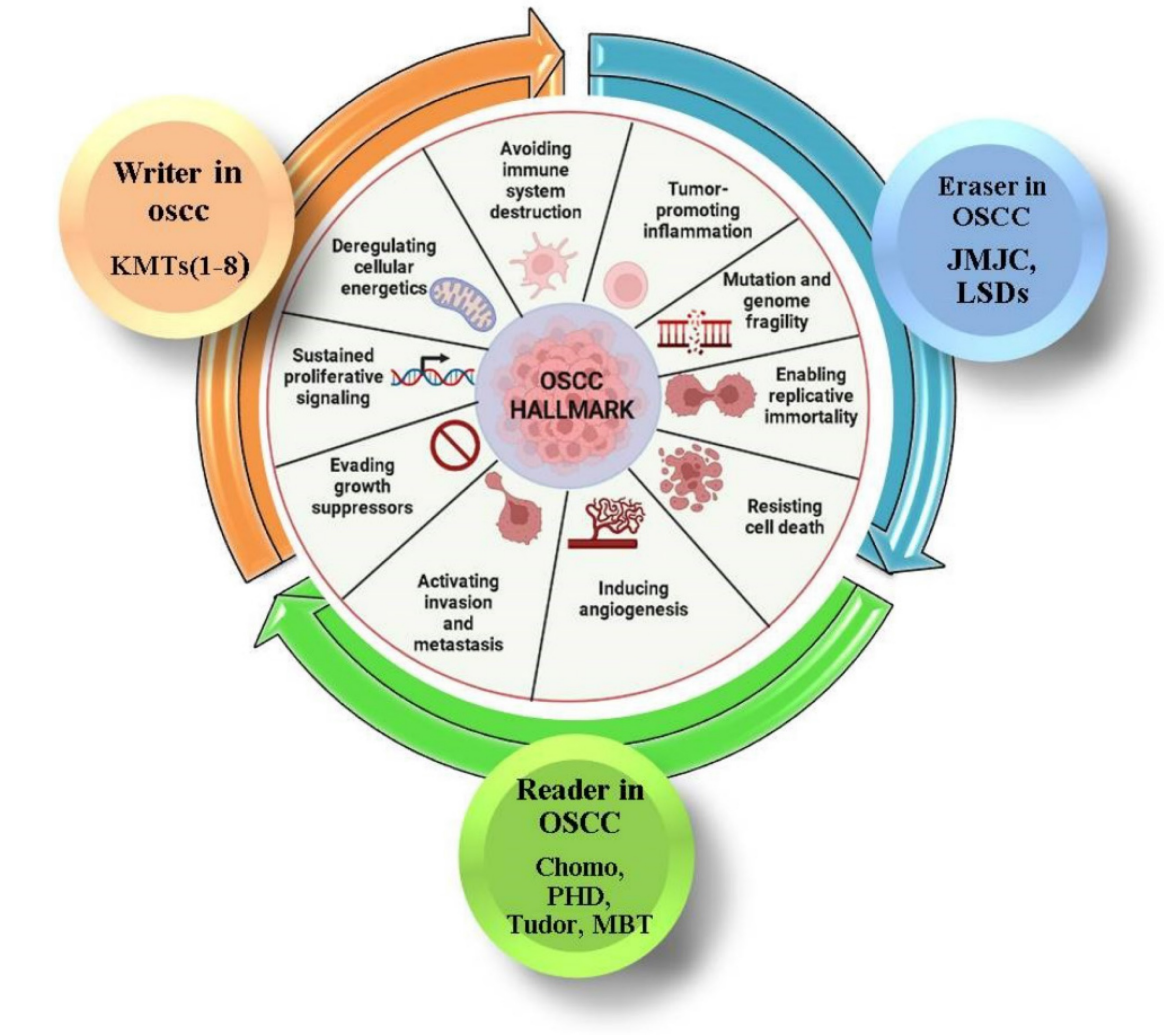
**Hallmark of OSCC and histone methylation.** This figure illustrates the role of histone methylation in oral squamous cell carcinoma (OSCC). Histone methyltransferases (writers, in orange), demethylases (erasers, in blue), and methyl-binding proteins (readers, in green) regulate chromatin structure, influencing gene expression and contributing to OSCC development. The inner circle represents key hallmarks of OSCC, such as avoiding immune destruction, promoting inflammation, and resisting cell death, all linked to histone modifications. The color coding represents the involvement of writers, erasers, and readers in these processes.

Histone methylations are present in several positions on the tail and globular domains of histones, and their stages are carefully regulated by methyltransferases, demethylases, and a variety of effector proteins. Writers and erasers are terms for enzymes that modify histones by adding or removing chemical groups. Reader proteins are able to recognise the altered histones. Depending on many factors, these histone modifiers can either promote or inhibit gene expression (Table 1) 47-54. Changes in the global levels of histone alterations in oral malignancies are related to a poor prognosis. Numerous studies have linked these methyltransferases, demethylases, and methyl-lysine-binding proteins to the development of OSCC (Figure 2) 54,55. As the tumour grows, abnormal cell production, invasion, and metastasis as well as chemoresistance might emerge from these changes in response to both internal and external stimuli. A variety of chromatin-modifying proteins that control the methylation of histones at specific locations, including H3K9, H3K4, H3K36, H3K79, H3K20, and H3K27, are important for OSCC. Thus, we focused on a set of histone methylation regulators that are crucial for OSCC.

## Histone methylation-writer in OSCC

The "writer" in histone methylation is thought to be the enzyme methyltransferase which mostly methylates side chain nitrogen atoms of lysine in histone H3 and H4. The addition of one, two, or three methyl groups from S-adenosyl-L-methionine to the -amino group of a histone lysine residue is catalyzed by these lysine methyltransferases (KMT1-6) to monomethylate, demethylate, or trimethylate a lysine residue 56. KMTs can be roughly divided into eight categories based on their structure and sequencing around the SET domain, with further subgroups including KMT1 (A-F), KMT2 (A-H), KMT3 (A-C), KMT4, KMT5 (A-C), KMT6, KMT7, and KMT8 57. The SET (suppressor of variant allele, enhancer of Zeste, trithorax) domain, which is a 130 amino acid conserved domain shared by all KMTs except DOT1L (disruptor of telomeres silencing-1) is responsible for methylating lysine 79 in the terminal domain of histone H3 to suppress variant allele. DOT1L methylates lysine residues in the globular core of the protein due to SET deficiency, as opposed to the tails of the histones 58, 14. Dot1 showed to be the first disruptor of telomeric silencing in *Saccharomyces cerevisiae* through a genomic screen. It is the only histone methyltransferase that is known because it lacks a SET domain. The methylation of H3K79 affects DNA repair, transcriptional control, a cell cycle checkpoint, telomeric silencing, and cellular development 59.

### Writer without SET domain

The DOT1L methyltransferase enzyme KMT4, part of the huge macromolecular complex known as DotCom (DOT1Lcontaining complex), was reported to be overexpressed and/or amplified in solid tumours like breast, ovary, prostate, and colon. It interacts with the chromosome 3 translocated mixed-lineage leukaemia proteins (MLLT3) to function even in OSCC. It is connected to the repair of UV damage since it is the primary enzyme for methylating the histone H3 lysine 79 (H3K79) 60,61. It influences the gene expression in humans, flies, and yeast 62, 63. H3K79 methylation controls cell invasion, proliferation, plasticity and stemness, cell cycle progression, cell-to-cell signaling, epithelial-to-mesenchymal transition, and chemoresistance through interactions with several molecular partners, including noncoding RNA 64. For example, the deregulation of HIF-1-dependent expression of genes like MMP1, MMP2, TWIST, VIM, and CDH1 in oral cancer is linked to the interaction of DOT1L proteins with MLLT3. This shows that one of the important factors in OSCC invasiveness may be MLLT3 linked with DOT1L. Further, DOT1L is required for MLLT3 to repress CITED4's transcription, which leads to the dysregulation of HIF-1-mediated genes and is allied to a poor prognosis for patients with OSCC 65. It also plays a substantial role in perinatal and embryonic development, which makes DOT1L an intriguing therapeutic target for oral cancer therapy 66.

### Writer with SET domain

SET domains are the cause of the enzymatic activity of other KMTs, including KMT1-3, 5, 6, and 7. Since most of their functions involved alteration in the ratio of gene expression through function gain or loss, increased expression, chromosomal translocation, downregulation by promoter hypermethylation, or mutations of histone modifying enzymes/complexes or even the histone modifying site are commonly detected in human cancers 67, 68. These enzymes also affect DNA damage response and polycomb silencing, both of which have a correlation to cancer. For instance, in the case of cancer, a high level of H3K27me3 was related to tumour progression (advanced T and N status, and stage of tumour) by preventing gene expression through the action of an enzyme called enhancer of zeste homolog 2 (EZH2/KMT6), which trimethylates histone H3 lysine 27 and subsequently causes transcriptional repression of target genes 39, 69, 70.

Oral cancer is more aggressive and has a worse prognosis when EZH2 is overexpressed because it induces self-renewal and prevents stem cells differentiating 71. Similarly, numerous studies showed that OSCC can upregulate EZH2 in cell lines 72-74. Additionally, EZH2 was linked to the expression of the putative G1 cyclin D1, the proliferation marker Ki-67, the tumour suppressor p53, the long non-coding RNA HOX transcript antisense RNA repressed E-cadherin, and the elevated receptor tyrosine kinase c-ros oncogene that stimulates cellular migration and proliferation 75-77. According to Zhao et al. 78, EZH2 plays a role in metastasis, invasion, metastasis, and cell proliferation in the development of OSCC. These results suggest that EZH2 is implicated in the development of OSCC. Strong EZH2 expression has a high correlation with the development of OSCC, with 80% of patients developing OSCC within five years, according to a study on oral leukoplakia (OL). In Leuk-1 cells, downregulation of EZH2 resulted in G1 arrest, decreased invasion, and downregulation of cyclin D1. These results point to EZH2 as a major participant in the malignant transformation of OL and as a possible biomarker for estimating the risk of OSCC 79.

Two H3K9 methyltransferases, GLP (also called EHMT1) and G9a (named KMT1C), form a heteromeric complex that is necessary to maintain the mono- and demethylation of H3K9, a feature of silent euchromatin. The processes of transcription, signal transduction, cell division, and proliferation depend on this system. The primary cause of transcriptional repression, according to Yokochi et al. 80 and Purcell et al. 81, is G9a's lysine methylation of histone and non-histone substrates. Effects of G9a expression on OSCC prognosis, clinical stage, and pathological grade 82. G9a expression was linked to invasion depth, lymph node metastasis, and tumor stage in 139 OSCC cases, demonstrating that it is necessary for reduced E-cadherin and methylation. G9a controls restrictive epigenetic modifications by mono- and di-methylating certain molecules. Inhibiting G9a with BIX in Tca8113 and KB cell lines, showed significant inhibition in cell growth and proliferation. Additionally, it promotes cell death through the production of cleaved caspase 3 and cell autophagy by the conversion of LC3-I to LC3-II. Thus, identifying G9a aberrations could be a promising epigenetic target for treating OSCC 41.

G9a promotes the features of cancer stem cells (CSCs) and the epithelial-to-mesenchymal transition (EMT) in HNSCC. G9a suppresses E-cadherin expression through its interaction with Snail, which promotes metastasis and poor prognosis. G9a knockdown highlighted the significance of the G9a-Snail axis in lymph node metastasis by reversing EMT, preventing cell migration, and lowering the expression of CSC markers. Treating metastatic HNSCC may benefit from a therapy approach that targets this axis 82. Other histone methyltransferases, such SUV4-20H1 and SUV4-20H2, catalyze the di- and tri-methylation of H4K20 at H4K20me2 and H4K20me3. These enzymes are also involved in DNA replication, cell cycle regulation, and DNA repair, among other biological activities. Similarly, the trimethylated SUV39H1 (also called KMT1A) enzyme regulates genes to produce autophagic cell death, which in turn promotes cell growth 42. Both Suv4-20h1 and Suv4-20h2 are amplified in a range of human malignancies, including those of the head and neck, bladder, breast, kidney, oesophagus, colon, uterus, lung, prostate, liver, and stomach, according to multiple studies employing the TCGA database. When compared to normal keratinocytes, Suv4-20h1 was significantly expressed in these cells, even in OSCC cell lines 83, 84.

These histone methylations at H3K9, H3K79, H3K20, and H3K27 demonstrate the connection between histone methylation and OSCC progression. Briefly, OSCC cells with various methylation patterns also had varied amounts of H3K4 and H3K9. In contrast to normal tissue, OSCC had lower levels of H3K4me3 and me2, whereas H3K4me2 levels were remained unchanged. The levels of H3K4me1 in the two types of tissues are not significantly different from one another. In H3K9, nuclear H3K9me2 function is associated with a poorer prognosis for both disease-free and disease-specific survival, whereas cytoplasmic H3K9me1 expression is associated with a lower probability of disease-specific death. Histone methyl transferases such as Prdm3 and Prdm16 (H3k9me1) in the cytoplasm create monomethylated H3 histone after pre-methylation of the histone. Methylated histones are believed to affect gene expression silencing and chromatin compaction. The SUV39h enzyme will convert H3K9me1 into H3K9me2 and H3K9me3 in these areas 85. Because of this, a tumor with higher levels of H3K9me1 expression in the cytoplasm is likely to have less histone methylation and a reduced chance of dying from the disease. These findings suggest that a regulatory role of methylation in altering chromatin structure, which may increase the risk for cancer 39, 86. Additionally, these epigenetic changes showed that these writer proteins can silence or activate key genes involved in chromatin remodelling, which possibly will lead to an open chromatin organization and enable transcription of elements associated with human malignancies such as OSCC.

## Histone methylation-eraser in OSCC

Histone demethylases (HDMs or KDMs) referred to be erasers that contribute to the elasticity of covalent histone alternations. This is because, they remove the mono-, di-, and tri-methylation marks in a reversible manner through S-adenosyl-L-methionine (SAM)-dependent mechanism 87. KDMs are diverse and can be divided into two main groups: histone demethylases with the highly conserved JumonjiC (JMJC) domain and lysine-specific demethylases (LSDs) of the amine-oxidase type. The first group family of demethylases, JMJC, can eliminate all three potential methylation states of methylated Lys residues, since the LSDs restricted the enzymatic activity to mono- and dimethyl Lys residues. The JmjC-KDM class, which is further subdivided into the KDM2/7, KDM3-6, subfamilies, contains enzymes that make up 28 of the 30 known KDMs 7, 87. Numerous biological processes, including cell cycle, ageing, DNA damage response, and heterochromatin formation, are influenced by KDMs. They reported to play a vital function in controlling pluripotency 88. KDM over-expression is necessary for a number of cancer types, and these functions are crucial for cell proliferation. Proto-oncogene and tumour suppressor gene expression levels can be changed by controlling the methylation of H3K9, H3K27, H3K4, H3K36, or other sites. Because they have a considerable impact on the expression of the majority of cancer-related genes, KDMs may be especially fascinating as drug targets. The involvement of histone lysine demethylases in the aetiology of HNSCC has made them intriguing molecular targets. Genomic analysis was used to find mutations in the KDM-4, KDM-5, and KDM-6encoding genes 89. KDMs were found to be overexpressed in OSCC, especially at advanced stages 90.

## Lysine Specific Demethylase (LSDs)

Our understanding of how histone methylation occurs during both normal biological processes and cancer was fundamentally altered by LSD1 (also known as KDM1A). This discovery led to the identification of over 20 histone demethylases that are currently known and to provide the first indisputable proof that histone methylation is dynamic. LSD1 is an essential component of several transcriptional co-repressor complexes, such as nucleosome remodelling and histone deacetylation and corepressor for element-1-silencing transcriptional factor, which selectively remove the methyl group from H3K4me1/2 and thereby mediate gene repression 91, 92. By genetically removing or pharmacologically inhibiting LSD1, cell migration, epithelial-to-mesenchymal transition, chemoresistance, and cancer stem cell characteristics were all effectively inhibited. In certain cases, antitumor immunity was also activated, which eventually prevented cancer growth and spread. This shows the substantial correlation between OSCC's aberrant, LSD1 overexpression, tumour aggressiveness and lower overall survival 93. Squamous cell carcinoma (SCC) metastasis, invasive growth, and the synthesis of AP-1 transcription factors were all aided by a unique LSD called KDM4A (lysine-specific demethylase 4A). KDM4A knockdown in an orthotopic nude mouse model of SCC, displayed dramatic reduced metastasis of SCC to cervical lymph nodes. In addition, compared to basic human SCC, KDM4A abundance was considerably higher in lymph nodes from human metastatic SCC. Thus, KDM4A might serve as a significant therapeutic target for preventing the spread of invasive SCC 94. Similarly, deregulation of the histone demethylase JMJD5 was found to have a significant impact on carcinogenesis.

## JMJC domain containing histone demathylase

JMJC domain-containing proteins have been linked to a number of malignant human cancers via epigenetic remodelling. The H3K36me2 demethylase formerly known as JMJD5 is now known as KDM8 95. In breast cancer 96, prostate cancer 96, and OSCC 97, JMJD5 is known to regulate the p53/nuclear factor-kappaB (NF-κB) pathway, the regulation of the cyclin A1 coding area, and as a dual coactivator of AR and PKM2 that integrates the AR/EZH2 network and metabolism of tumour. In OSCC cells, JMJD5-knockdown increased the expression of E-cadherin while decreasing the expression of N-cadherin and vimentin. The enhanced cleavage of Caspases and PARP-1 provided additional proof that JMJD5-silence triggered apoptosis through both intrinsic and extrinsic routes. Meanwhile, JMJD5-knockdown also increased p53 expression levels. In JMJD5-silenced cells, inhibiting p53 expression with its inhibitor PFT prevented the apoptotic response. Pifithrin (PFT) pre-treatment prevented the NF-κB reduction caused by JMJD5 inhibition. JMJD5 overexpression consistently decreased levels of p53, cleaved caspase-3, and PARP-1 in OSCC cell lines while increasing nuclear NF-κB expression. These results support the function of JMJD5 to serve as a critical prognostic indicator and therapeutic target for halting OSCC progression 97, 98.

Similarly, tumour suppressor gene silencing in patients with oral and oropharynx cancer is associated with the JMJD1A protein's promotion of histone demethylation, particularly at lysin-9 of H3K9me2 or H3K9me1 histone H3. Adrenomedullin (ADM), a JMJD1A gene target, has been found to induce carcinogenesis and cell proliferation. JMJD1A and ADM expression, together with the amount of H3K9 methylation, have all been connected to the risk of developing and the prognosis of HNSCC as potential indicators of prognosis in this malignancy 99. JARID1B, referred as PLU-1, is a homolog of jumonji, AT rich interactive domain (JARID) and Retinoblastoma-Binding Protein 2 (RBP2). It has H3K4 demethylase activity and belongs to the JARID family 100. Some studies indicate that OSCC is one of the cancers where JARID1B is overexpressed. In all of these cancers, JARID1B expression is associated to higher metastases and mortality rates, but JARID1B knockdown causes cell death through cell cycle arrest 101-103. Even RNA demethylase (ALKBH5) affects HNSCC in an oncogenic manner. *In vitro* and *in vivo* tumour growth is suppressed by RNA demethylase (ALKBH5) silencing of m6A demethylase alkB homolog 5. The m6A modification of DDX58 mRNA is downregulated by ALKBH5, according to m6A-RNA immunoprecipitation sequencing. Because of the ablation of RIG-I expression brought on by ALKBH5 overexpression, the inhibitor of NF-κB kinase ε/TANK-binding kinase 1/interferon regulatory factor 3 (IKKε/TBK1/IRF3) pathway is blocked, which ultimately promotes immunological escape and reduces IFNα release. This showed that RIG-I-mediated IFNα secretion is blocked by ALKBH5 overexpression through the IKKε/TBK1/IRF3 pathway, suggesting a new mechanism governing IFNα production in the tumor microenvironment 104 Since histone demethylase affects OSCC development modulation, these above enzymes might be considered as crucial prognostic markers and therapeutic targets for OSCC progression.

## Histone methylation-reader in OSCC

In order to accommodate a modified histone residue, readers also known as polycomb-group (PcG) proteins often offer an accessible surface, such as a cavity or groove. They are also able to identify the precise state, such as mono- or trimethylation of lysine, or the modification, such as acetylation or methylation. In order to ascertain the context of the sequence, reader proteins additionally interact with the changed amino acid's flanking sequence. These reader proteins have variety of methyl-lysine-binding motifs, including the ADD, tudor, WD40, BAH, ankyrin repeat, zn-CW, MBT, BMI-1, chromo, PWWP, and PHD domains. Target methyl-lysines can also be distinguished based on their amino acid sequences and the amount of methylation around them 105. Recent research has suggested that the PcG proteins are important components of an epigenetic memory system that controls overall gene expression during development in multicellular eukaryotes 106. The Polycomb repressive complexes (PRCs-PRC1 and PRC2) of PcG proteins are multiprotein repressive complexes that restrict transcription by a mechanism that includes chromatin remodelling. PRC1 recruits other components and binds to this histone mark (H3K27me3), which causes chromatin compaction and epigenetic silencing of the nearby genes by ubiquitinating a lysine residue of histone H2A. Additionally, the PRC2 trimethylates histone H3 Lysine 27 (H3K27) is recruited to DNA sequences like Polycomb response elements that include certain cis-regulatory elements 107. According to recent research, these proteins are crucial for the development and spread of cancer in humans as well as human carcinogenesis. The dysregulation and malfunction of PcG proteins frequently showed to resulted in the blocking or improper activation of developmental pathways, the promotion of cellular proliferation, the inhibition of apoptosis, and the expansion of the cancer stem cell population 108. BMI-1 for instance, controls a variety of biological processes, including X chromosome inactivation, carcinogenesis, and stem cell renewal. It is one of the key components of the polycomb repressive complex 1 109. The ink4a and ink4b loci, which encode the proteins p16INK4A, p19ARF, and p15INK4B, are known to be the cellular target genes of B-lymphoma MO-MLV insertion region-11 homolog (BMI-1) 110.

The ink4a locus, which is crucial for the beginning of cellular senescence in a variety of human somatic cell types is suppressed by BMI-1, which is thought to encourage cellular proliferation 111. Additionally, the abrogation of the p16INK4A/pRb pathway in neoplastic cell line derived from human oral keratinocytes (HOK-16B-BaP-T cells) caused BMI-1 knockdown, which resulted in a fast arrest of replication and the death of feasible cells. These findings show role of BMI-1 to control cellular proliferation during the development of oral cancer by p16INK4A-independent pathways 112. Human cancers are known to exhibit abnormal expression, inactivating mutations, chromosomal translocations, and recruitment of PcG proteins and complexes by methylation histone. The frequency of these recently discovered mutations and abnormalities shows the essential of dysregulation of PcG activity for the biology of cancer stem cells and cancer formation. The abundance of PcG complexes and the many PcG proteins inside these complexes make it difficult to draw a precise picture of the precise regulatory events involving PcG complexes in OSCC and other cancers. To improve the therapeutic potential of these proteins, more studies are needed to address this problem 113.

## Personalised medicine

The epigenetic principles illuminate the path for the rapidly developing field of personalised medicine, which uses diagnostic tests based on genomic, proteomic, and metabolomic data to improve and predict patient responses to targeted therapy. Additionally, the combination of the human genome, information technology, biotechnology, and nanotechnology to provide therapy based on distinct differences compared to population trends is opening up new avenues for the rapid detection of biomarkers for oral cancer 114. Likewise, personalised medicine that could be applied to personalised dentistry facilitates reducing high-risk invasive testing procedures, facilitating to control overall health care costs, decreasing adverse drug reactions, directing targeted therapy, and reducing trial-and-error procedures as well as to increase patient willingness to treatment 115. These epigenetic phenomena that link genetics, nutrition, and environment have an impact on oral cancer 116, 117.

The majority of these chromatin writers, readers, and erasers are good candidates for therapeutic management. As previously mentioned, the improper writing, erasing, or reading of the histone alteration language inherent in chromatin signifies a frequent, often early, and significant contribution to the oncogenesis of OSCC through the generation of epigenetic, transcriptomic, and phenotypic mutations. The treatment of OSCC as personalised medicine will be improve A better understanding of various biochemical mechanisms underlying the dysregulation of this chromatin language in oral cancer, as well as the identification and improvement of more potent drugs to target these chromatin-related vulnerabilities, 118. Early detection is critical in this case because only one-third of OSCC patients had the disease in stages I and II of diagnosis 119. Furthermore, there is a wide variation in the efficacy of currently available treatments for oral cancer from patient to patient. They frequently harm healthy, non-cancerous organs and tissues. Personalised medicine may make use of histone methylation to focus specific therapies to certain organs, gene changes, and unique traits relevant to each individual case of cancer 120. Histone methylation can be a target for drugs since it is chemically reversible. Small-enzymatic inhibitors that work to restore the aberrant epigenetic machinery have the potential to reverse epigenetic markers in cancer 121. As previously stated, controlling histone methylation has significant clinical potential, particularly for oral cancer treatment regimens. It is now evident that histone methylation has the potential to be an epidrug that targets using small enzymatic inhibitors, much like a personalised treatment. Particularly, in the current study, we discussed how lysine methylation (writer, eraser, and reader) influence cell migration, proliferation, and cell cycle progression, as well as certain characteristics of cancer stem cells in OSCC.

## Lysine methylation as biomarker in OSCC

Histone methylation significantly influences the epigenetic regulation of gene expression during a number of biological processes, including cellular stress response, cell cycle control, embryogenesis, DNA damage response, and cell differentiation 122,123. In addition to genetic changes, these epigenetic tumor-specific changes, like lysine methylation, are detectable in circulating tumour cells (CTCs) or circulating free tumour DNA (ctDNA) using liquid biopsy assays in plasma or other body fluids. These biomarkers have demonstrated diagnostic, prognostic, and predictive abilities 124,125. Due of their impact on tumour characteristics, including differentiation, apoptosis, and treatment response, these histone methylation alterations are particularly associated to cancer. Histone H3 methylation at residue 9 (H3K9) is especially linked to transcriptional repression because it induces the development of heterochromatin and silences tumour suppressor genes in a variety of cancer types 126. For instance, Western blotting investigations were carried out employing particular antibodies to assess levels of histone methylation (H3K4). The findings suggest that almost all modifications involving the methylation of histone lysine residues are characterized by a reversible process. By comparing the band strength of the examined samples with the bands visible in the histone preparation from HeLa cells on the same films, the methylation levels of H3 histone are expressed in arbitrary units (A.U.). This implies that histone methylation may act as a biomarker in OSCC and be beneficial in regulating the oncogenic nature of OSCC cells. Similarly, when compared to the healthy oral mucosa, OSCC and leukoplakias have the similar H3K4 methylation behaviour. Relative to healthy mucosae, they both exhibit greater demethylation and a more pronounced decreased trimethylation level. In contrast, only carcinomas did not significantly differ from healthy mucosae in terms of monomethylating levels. Tumorigenesis has been linked to a considerable alteration in the levels of the heterochromatin markers H3K9me3 and H4K20me3, respectively 127. The JMJD2C/GASC1 gene is also amplified in OSCC 128. These results demonstrate that histone methylation expression may serve as potential treatment targets as well as prediction markers of oral cancer progression and prognosis. Therefore, abnormal regulation of these methylation marks may contribute to the carcinogenic potential and may also serve as a therapeutic target.

## Lysine methylation related therapeutic targets

Recent advances in protein research have made it possible to identify lysine-methylated proteins, and it has been estimated that the human proteome contains about 1200 different proteins with a total of about 2000 lysine residues with methyl modifications. The majority of these methylation's biological purpose has yet to be discovered. These post transcriptional modifiers that control the activity of promoter-specific transcription factors are a major mediator of the dynamic changes in gene expression that happen in response to external cues 129. Lysine methylation is emerging as a major regulatory mechanism of transcription factor function because the regulation of these changes activates or represses gene expression 130. This methylation status of gene promoters and their relationships to clinical indicators in patients with solid tumours and haematological malignancies have been the subject of numerous researches as mentioned above. These analyses have revealed some connections between lysine methylation and survival or therapeutic response, changes in the epigenome of cancer cells, and clinical survival indicators. Lysine methylation specifically influences DNA-binding affinity, protein-protein interactions, protein stability, subcellular localization, and cell signalling to regulate the function of transcription factors (TFs). Changes in lysine methylation can also have an impact on the position of transcription factors in the nucleus, which in turn regulates transcriptional activity. This is significant since these changes can alter how physiologically active cells perform. Additionally, it facilitates the affinity with which transcription factors bind to their targets, altering the transcription of their target genes and eventually the way in which proteins interact. Thus, histone methylation caused by a gene mutation, translocation, or overexpression can often be the first sign of an illness like cancer.

Identification of genes whose transcriptional suppression affects sensitivity to antineoplastic treatments is crucial for individualised anticancer therapy. It is also critical to convert this data into practically applicable assays that will direct anticancer therapies. Additionally, gene profiles will be required to help each patient specifically, but such tests are not yet accessible. Since this region is a possible pharmacological target, small molecule inhibitors of these proteins are helpful chemical probes or prospective therapies. In contrast to HDAC inhibitors, the development of histone methylation modulators is still in its early stages 131. There were few potent HMT and KDM inhibitors available before 2010. Since a couple of years ago, researchers and the pharmaceutical sector have greatly increased their efforts, leading to a rapid increase in the number of small molecule histone methylation modulators 54.

Tazemetostat has been utilized at concentrations between 0.1 µM and 10 µM in *in vitro* experiments. Additionally, additional HMT inhibitors often have dosages between 5 µM and 25 µM, which corresponds to the different susceptibilities of different cancer cell lines to these compounds 132. For instance, G9a is dysregulated in a number of malignancies, such as leukaemia, ovarian, lung, and breast tumours. Although cellular toxicity hindered its usage in clinical settings, the first-identified G9a inhibitor, BIX-01294 suppressed tumour growth in mice xenograft models of breast, hepatocellular, and OSCC. Later, additional BIX-01294 variants were created 122, 56. Similarly, the polycomb repressive complex 2 (PRC2) suppresses gene expression and methylates histone H3 lysine 27 to control cell division and proliferation. Enhancer of zeste homolog 2 (EZH2) or EZH1, a near homolog, are found in two distinct PRC2 complexes and serve as a kinase domain of PRC2. EZH1/2 dual inhibitors were more effective at reducing histone H3 lysine 27 trimethylation in cells than EZH2 selective inhibitors. In comparison to an EZH2 selective inhibitor, higher anticancer activity were demonstrated against diffuse large B-cell lymphoma cells containing EZH2 gain-of-function mutations 133, 134. Some cancer cell lines were susceptible to the EZH1/2 dual inhibitor both *in vitro* and *in vivo*. In drug concentrations greater than those employed in the anticancer investigation, rats treated with EZH1/2 dual inhibitors for 14 days did not exhibit any evidence of severe toxicity. These results imply that EZH1/2 dual inhibitors may have clinical applications in solid tumours like oral cancer 135,136.

## Conclusion and future hopes

**Figure 3:**
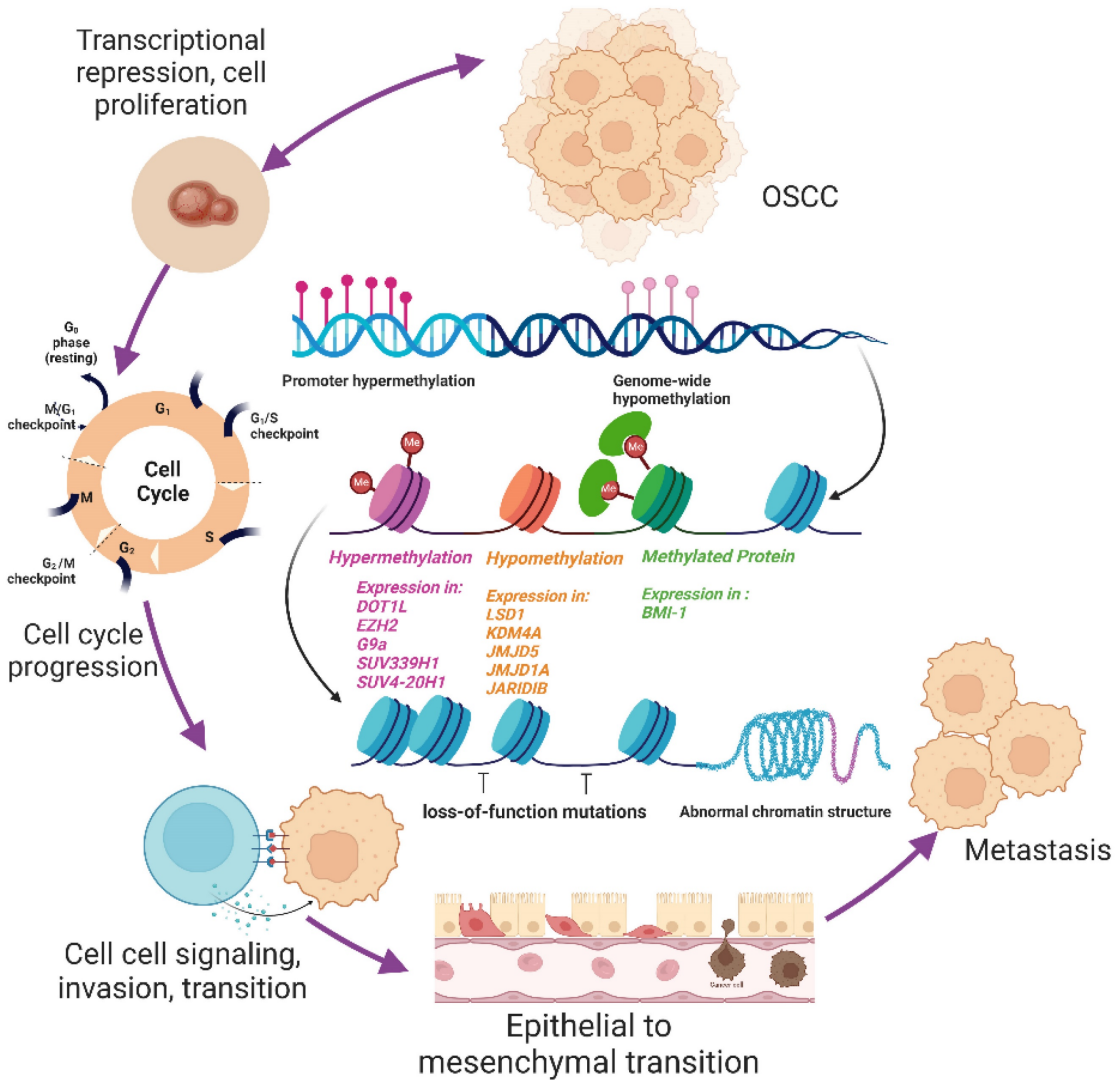
** OSCC progression in relate to histone methylation.** Figure 3 depicts the transition of healthy oral squamous cells into oral squamous cell cancer cells in accordance with the histone methylation mechanism. Histone methylation modifiers like "writer," "eraser," and "reader" can alter the cell cycle, which regulates cell signalling, proliferation, invasion, and migration that results in the epithelial to mesenchymal transition. This alteration may result in metastasis, additional signalling activation, the development of cancer stem cells, or chemoresistance.

OSCC is a serious public health issue globally due to high mortality and poor prognosis. The most frequent reasons of death in OSCC patients include second primary tumours, lymph node invasion, and high rates of radiation and chemotherapy recurrence. Methylation, histone modifications, and non-coding RNA are among the epigenetic components that have recently provided compelling evidence that genetic and environmental factors are the primary causes of oral cancer's onset and progression. Genome editing during the cell cycle has a significant impact on histone modification at the chromatin level. Depending on many factors, these histone methylation proteins, such as histone methyltransferase, histone methylation detecting proteins, and histone methylation-regulatory proteins, can either promote or inhibit gene expression. It can regulate post-transcriptional repression, cell migration, invasion, migration, cell progression, epithelial-mesenchymal transition, and chemoresistance. The current review emphasizes the significance of histone methylation in OSCC as a biomarker and therapeutic target, which opens up the possibility that it will potentially serve as a tool for personalised therapy (Figure 3). The identification and progress of more effective drugs to target dysregulated histone expression would enhance the treatment of OSCC as individualized medicine. Because oral cancer treatments that are currently available have a very variable degree of effectiveness from patient to patient and frequently harm healthy, non-cancerous organs and tissues. In personalized medicine, histone methylation may be utilized to direct specific treatments towards target organs, gene alterations, and other factors relevant to each unique case of cancer.

Being the most common type of cancer in the world, OSCC is epigenetically controlled by the transcription of genes involved in cancer cell migration, invasion, cell death, and proliferation. It is clear that this epigenetic mechanism may be used in the future to improve the development of the treatment of oral cancer through individualized therapy. Utilizing cutting-edge technology like the CRISPR/Cas9 system, which has tremendous efficacy in discovering genes linked with oral cancer pathobiology and in treating the same by gene deletion technique, histone methylation has to be studied more thoroughly in OSCC as the future hope. The histone methylation study will be a triumph in oral oncology for early detection, diagnostics, and therapeutic components that help in identifying person's genetic profile, disease prediction in its early stages, and applications in personalised medicine.

## Figures and Tables

**Table 1:** Overview of research on histone modifications in Oral Squamous Cell Carcinoma (OSCC) and their mechanistic implications

S.No	Study Type	Histone Modification	Key Findings	Outcome	Ref
1	*In vivo* (tissue)	H3K4Me1, Me2, Me3	Altered levels of H3K4Me2 (↑) and H3K4Me3 (↓).	May contribute to oncogenesis.	47
2	Human study	H3K4ac, H3K27Me3	Advanced tumors linked to low H3K4ac and high H3K27Me3.	Predictive of OSCC outcomes.	48
3	*In vitro*	H3K9 trimethylation	G9a inhibition suppressed cell growth and induced cell death.	Reduced colony formation.	49
4	*In vitro* & *In vivo*	H3K4Me3	Regulates Wnt/β-catenin signaling and stem cell programs.	Linked to transcriptional activation.	32
5	*In vitro*	H4K20Me2	SET knockdown increased H4K20Me2 and miR-137 levels.	Regulates miRNA production in OSCC.	50
6	*In vitro* & *In vivo*	KMT2D	Knockdown reduced tumor growth and Wnt signaling.	Impairs OSCC progression.	14
7	*In vitro* & *In vivo*	H3K27ac, H3K27Me3	Metformin inhibited cell proliferation and colony formation.	Induces apoptosis in OSCC cells.	51
8	*In vitro*	DNMT, HMTi, HDACi inhibitors	Combined treatment reduced cell viability and induced apoptosis.	Cell cycle arrest at S and G2/M phases.	41
9	*In vitro*	Various histone markers	Altered markers linked to homeobox gene dysregulation.	Associated with OSCC development.	52
10	*In vitro*	H3K36 methylation	Impaired methylation prevents differentiation.	Promotes oncogenesis in HNSCC.	53
11	*In vitro*	DOT1L	DOT1L upregulation linked to cancer stem cell (CSC) invasion and cisplatin resistance.	Promotes survival proteins via miR-10.	54
